# Complementary actions

**DOI:** 10.3389/fpsyg.2015.00557

**Published:** 2015-05-01

**Authors:** Luisa Sartori, Sonia Betti

**Affiliations:** ^1^Dipartimento di Psicologia Generale, Università di Padova, Padova, Italy; ^2^Cognitive Neuroscience Center, Università di Padova, Padova, Italy

**Keywords:** action observation, perception–action coupling, social interactions, motor resonance, transcranial magnetic stimulation

## Abstract

Complementary colors are color pairs which, when combined in the right proportions, produce white or black. Complementary actions refer here to forms of social interaction wherein individuals adapt their joint actions according to a common aim. Notably, complementary actions are incongruent actions. But being incongruent is not sufficient to be complementary (i.e., to complete the action of another person). Successful complementary interactions are founded on the abilities: (i) to simulate another person’s movements, (ii) to predict another person’s future action/s, (iii) to produce an appropriate incongruent response which differ, while interacting, with observed ones, and (iv) to complete the social interaction by integrating the predicted effects of one’s own action with those of another person. This definition clearly alludes to the functional importance of complementary actions in the perception–action cycle and prompts us to scrutinize what is taking place behind the scenes. Preliminary data on this topic have been provided by recent cutting-edge studies utilizing different research methods. This mini-review aims to provide an up-to-date overview of the processes and the specific activations underlying complementary actions.

## Introduction

*Motor resonance* is defined as the subliminal activation of the motor system—and of the *imitative* response—while observing actions performed by others (reviewed in Heyes, 2011). Gallese (2001) explained that: “when we observe actions performed by other individuals our motor system ‘resonates’ along with that of the observed agent” (pp. 38–39). Numerous neurophysiological studies have in fact demonstrated that a motor resonant mechanism is at work in the motor, premotor, and the posterior parietal cortices when individuals are instructed to observe goal-directed actions being executed by another or others (for review, see Fadiga et al., 2005; Heyes, 2011; Rizzolatti et al., 2014). The discovery of mirror neurons in monkeys provided the physiological model for this perception–action coupling mechanism (Rizzolatti and Craighero, 2004). Located in the ventral premotor cortex (area F5) and the posterior parietal cortex, mirror neurons were found to fire *both* when a monkey carried out a goal-directed action as well as when it observed that same action being performed by another subject (di Pellegrino et al., 1992). Motor resonance appears then to pre-activate the motor system of an observer in order to represent and interpret the movements of another person even before the “go” signal has been given and activation remains for the most part on an unconscious level (Costantini et al., 2011).

While actions that are observed and those that are being planned appear functionally equivalent (Knoblich and Flach, 2001), it is unclear if the visual representation of an observed action inevitably leads to its motor representation. This is particularly true with regard to *complementary* (from Latin *complementum*; i.e., that fills up) actions, a specific class of movements which differ from -although interacting with- an observed action (Sebanz et al., 2006; Knoblich et al., 2011). In the case, for example, that someone hands us a mug by its handle, we will automatically, without giving it a second thought, grab the mug using a whole-hand-grasp (the most appropriate grasping posture in this particular situation). The types of grasps adopted by the two interacting agents are incongruent, but they are nevertheless appropriate and complementary.

As a working definition, complementary actions refer here to any form of social interaction wherein two (or more) individuals coordinate and mutually complete their incongruent actions, rather than performing imitative behaviors. In this respect, we can define as *complementary affordances* all the action possibilities in which suitable motor programs aiming to bring a joint goal to completion are activated (such as grasping and offering a coin when seeing an open hand in sign of request). Depending on its posture and context, therefore, an extended open hand could lead to a donation, to a handshake or to an infinite number of other actions (Sartori et al., 2009). Activation of a complementary affordance is an important social tool, and it suggests that the automatic, rapid decoding of social cues influences intentional behavior in our everyday interactions, maximizing the efficiency of our responses. These examples illustrate the functional importance of complementary actions in the action–perception domain (Graf et al., 2009), and they prompt us to examine the mechanisms involved in producing those responses.

## Behavioral Studies of Complementary Actions

Since the direct matching between observed and performed actions is thought to occur automatically, when we observe an action which differs from our intended action we have to inhibit the tendency to imitate (Brass et al., 2005). While the mechanism leading to automatic imitation is relatively well-studied (Heyes, 2011), it is less clear how this automatic tendency is brought under control.

Evidence that task representation plays a pivotal role in shaping our actions has been provided by a series of studies (Newman-Norlund et al., 2007a,b; van Schie et al., 2008b; Poljac et al., 2009) in which participants were explicitly instructed to prepare imitative or complementary actions after viewing a virtual actor grasp a manipulandum using either a precision grip (PG; i.e., opposition between the index finger and thumb) or a whole-hand grasp (WHG; i.e., opposition of the thumb with the other fingers). As expected, participants were faster at preparing their response in imitative contexts if the action to be carried out was congruent with what they had observed. When, instead, they were expected to carry out complementary actions, they responded faster when their action was dissimilar to the one they had just observed. The task representation (imitative vs. complementary) seems then to overrule long-term stimulus-response associations, influencing the way that action–perception coupling takes place. Further evidence concerning this flexible perception–action coupling was produced by a 3D motion capture study (Ocampo and Kritikos, 2010) in which reaching and grasping parameters of congruent responses were found to improve in imitative contexts, and incongruent responses were facilitated in complementary contexts. Consistent with these findings, Longo et al. (2008) demonstrated that also the level of action coding can be modified (e.g., toward coding in terms of movements) depending on task requirements. Taken together, these data challenge the idea that action observation automatically leads to imitation in the observer and suggest that, depending on the context, observed actions can prime incongruent responses.

Recently, Sacheli et al. (2012, 2013) showed that participants involved in face-to-face interactions can mutually adjust their movements in time and space even in the absence of instructions to either imitate or perform a complementary response. This demonstrates that priming does not strictly depend on task-constrains, and that humans might indeed be able to actively shift from imitative to complementary actions, thanks to neuro-cognitive processes that still needs to be clarified.

## Neuroimaging Studies of Complementary Actions

Few studies have examined the neural circuitry behind joint actions, and in particular the human mirror neuron system’s (hMNS) involvement in complementary forms of social interaction. Might the hMNS provide a substrate for complementary actions? And if not, what role do other brain systems play?

**FIGURE 1:**
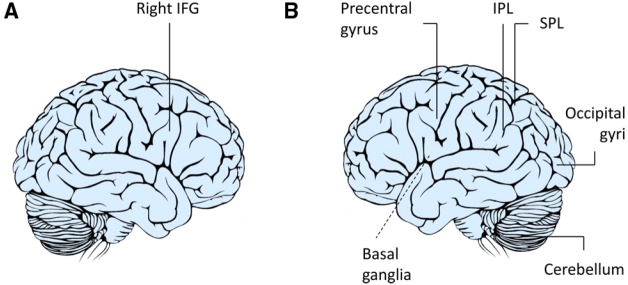
**Neuroimaging studies of complementary actions.** A number of studies have suggested that the right IFG **(A)** is not only involved when we respond to the actions of others by doing the same as they do (imitation) but also when responding with complementary actions (Newman-Norlund et al., 2007a,b, 2008; Ocampo et al., 2011; Shibata et al., 2011). In contrast, others hypothesize that the flexibility required during complementary actions requires a large network **(B)** including the IFG, IPL, superior parietal lobule (SPL), precentral gyrus, basal ganglia, middle and temporal occipital gyri, and cerebellum to be involved in integrating one’s own actions to those of others (Kokal et al., 2009; Kokal and Keysers, 2010).

In a pioneering experiment, the response of the hMNS was specifically investigated in imitative and complementary action contexts using functional magnetic resonance imaging (fMRI; Newman-Norlund et al., 2007a,b). Signals were recorded while the participants prepared to grasp a manipulandum in one of two ways—with a WHG or a PG—after they viewed an actor carrying out that action. It was found that preparation for complementary actions resulted in an increased blood-oxygen-level-dependent (BOLD) signal in the right inferior frontal gyrus (IFG) and in the bilateral inferior parietal lobule (IPL), two core components of the mirror system (Figure 1). This finding can be explained in terms of *different kinds* of mirror neurons: strictly congruent mirror neurons, which respond to identical actions, both observed and performed ones, and broadly congruent mirror neurons, which respond to non-identical observed and performed actions and objects linked to them (Fogassi and Gallese, 2002). It is also possible that in the complementary condition, when participants observe an action drawing attention to an object eliciting a different action, an interplay takes place between mirror and canonical neurons with the latter responding both during the time the action is being executed and also while the objects linked to those behaviors are perceived (Rizzolatti and Craighero, 2004). The need to carry out a complementary action involving a different object might then imply a combination of mirror and canonical neurons coding for different types of actions at different times of the sequence. The hypothesis that different classes of mirror neurons serve to integrate observed and executed actions during complementary kinds of social interaction is certainly an appealing one.

Newman-Norlund et al. (2007a,b, 2008) also hypothesized that a joint action could preferentially recruit right lateralized components of the mirror system since right inferior frontal activations are linked to inhibition processes (Brass et al., 2005). Planning and executing complementary actions in this framework would mean, first of all, actively inhibiting the natural tendency to imitate observed actions. In the light of recent debates revolving around mirror mechanisms (Gallese and Sinigaglia, 2011; de Bruin and Gallagher, 2012), some have theorized that mirror neurons transform perceptual information regarding an intentional action in terms of the observer’s own action possibilities (Gazzola et al., 2007). The idea that the hMNS could link perceived actions with appropriate motor plans was confirmed by an fMRI study designed by Ocampo et al. (2011) who studied the neural activations underlying execution of actions that were unlike the ones observed. As expected, activity within the right IPL and right IFG—core regions of the hMNS—was greatest in the imitative context when the participants responded with actions that were similar to the hand actions observed. Interestingly, activity within these regions also increased when dissimilar actions were performed, indicating that there are increased demands linked to remapping stimulus-response associations (Figure 1A). Shibata et al. (2011) likewise found that the right IFG was involved in mediating higher-order action understanding linked to a complementary action request. Overall, these findings seem to suggest that there are two separate processes and that both are supported by fronto-parietal brain regions. The first process operates at a simple motor level within contexts that require similar responses. The second allows the observer to inhibit those responses and to prepare an action that is compatible with the task demands at hand.

A more integrated description of neural circuits underlying complementary actions was recently outlined by Kokal et al. (2009; Kokal and Keysers, 2010; Figure 1B). Participants in an interactive fMRI study were instructed to carry out complementary and imitative actions in real-time cooperation with an experimenter (“Joint Action”), by performing the same actions individually (“Execution”), or by simply observing the experimenter’s actions (“Observation”). This experiment raised our understanding of social interactions to an entirely new level by specifically mapping the contribution of the hMNS (i.e., common voxels for both execution and observation) as well as the areas specifically involved in the joint actions (i.e., voxels exceeding the sum of execution and observation). The areas responsible for this integration process were located bilaterally in the IFG, IPL, precentral gyrus, superior parietal lobule, middle and temporal occipital gyri, and cerebellum.

Two anatomically separate networks have thus been delineated: one that would decipher observed and executed actions into a single common code (Etzel et al., 2008) and another that would integrate this information to successfully achieve common goals. These findings show that although the hMNS plays a critical role in translating all actions into a common code, their flexible remapping seems to be performed elsewhere. It would seem then that any potential discrepancy between an observed action and a complementary response can be resolved flexibly in a two-step manner. During the first step, the observed action is processed in order to predict its goal. During the second, associations are made between the action observed and the appropriate response needed to accomplish a complementary goal. Crucially, Erlhagen et al. (2006) proposed an anatomical model based on animal studies differentiating direct (automatic) and flexible routes for action–perception coupling. The model involves four interconnected brain areas, namely the superior temporal sulcus (STS), area PF (Brodmann area 7b), area F5, and the prefrontal cortex (PFC). The STS-F5 connection, allowing for the matching between a visual description of an action and its motor representation, would represent the neural basis of the direct route for the automatic imitation of an observed action. More importantly, when required, the flexible action–perception coupling is realized in the model by the connection between the PF area and the PFC through which goal representations from the PFC can modulate and set the coupling between visual (STS) and motor (F5) representations (Erlhagen et al., 2006).

Notably, the temporal course of the low- and high-level systems interaction has long been debated.

If output from control systems guide and modulates the mirror system, this would represent a top-down process. The STORM model (social top-down response modulation) suggests that the decision to imitate or to inhibit imitation initially draws on social signals and is most likely supported by a brain network including medial Prefrontal Cortex (mPFC) and temporoparietal junction (TPJ), two core areas of the so-called Mentalizing system, engaged when participants judge other people’s mental state (Wang and Hamilton, 2012; Hamilton, 2015). Recently, Cross et al. (2013) have proposed a model for conflict processing in case of incongruence between observed and executed actions. When preparation to avoid imitation is not possible, medial prefrontal regions (mPFC and anterior cingulate cortex) would first detect imitative conflict and send information to anterior insula which would process conflict resolution, suppressing the unwanted motor activation. The hMNS would be therefore the target of top-down mechanisms of conflict resolution. In contrast, if the hMNS leads to an automatic tendency to imitate an observed action and this information feeds up to a monitoring system, this represents a bottom-up process (Brass et al., 2009). According to Ubaldi et al. (2015; see also Barchiesi and Cattaneo, 2013), early mirror responses (150 ms from onset of visual stimuli) would be followed by later rule-based non-mirror responses (300 ms). These data seem to indicate that a fast bottom-up process mediated by the dorsal visual stream produces automatic imitative responses. Whereas rule-based visuomotor associations would be mediated by a slower top-down system, relying on the PFC.

## Neurophysiologic Studies of Complementary Actions

**FIGURE 2:**
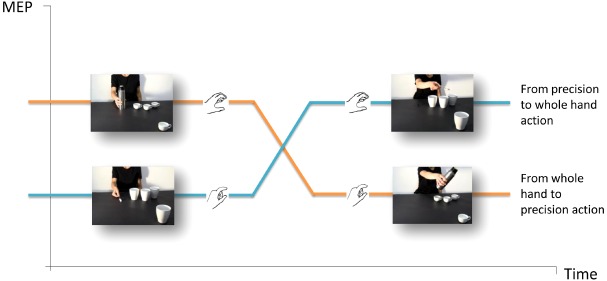
**The functional shift.** A fundamental requirement for successful complementary actions is the capacity to smoothly and efficiently switch from observing another person’s gestures to planning a corresponding reciprocal action. TMS-induced MEPs were recorded from participants’ hand muscles in response to observing an actor grasping an object and then trying vainly to fulfill a task (e.g., pouring coffee) in a cup which was strategically placed out of her reach but in the video foreground, close to the observer’s right hand (Sartori et al., 2012, 2013b,c). The type of grasp observed and the one that was required were reciprocally mismatched in all the videos (i.e., a WHG performed by the actor vs. a PG requested of the observer, and *vice versa*).

Action observation automatically activates corresponding motor representations in an observer, and the stronger support for this process comes from single-pulse transcranial magnetic stimulation (spTMS) over the primary motor cortex (M1) and concomitant electromyography (EMG; e.g., Fadiga et al., 1995). This technique allows to measure modulations in an observer’s cortico-spinal (CS) excitability while he/she watches an agent performing an action. A statistically significant increase in TMS-induced motor evoked potentials (MEP) amplitudes in the corresponding muscles indicates that observers are specifically attuned to the observed action and at what time it does occur. The facilitation of CS excitability provided the first physiological evidence for a direct matching in humans between action perception and action execution (for review, see Fadiga et al., 2005), and made it possible to explore motor system reactions in interactive contexts. A series of recent neurophysiologic studies were designed to assess the facilitation of CS excitability while participants observed video-clips evoking imitative and complementary gestures (Sartori et al., 2011b, 2012, 2013a,b,c). In one of these studies (Sartori et al., 2012), TMS-induced MEPs were recorded from the participants’ hand muscles while they observed an actor grasping an object and then unsuccessfully attempting to complete a task (e.g., pouring coffee in a cup which was strategically placed out of her reach but in the video foreground, close to the observer’s right hand). An almost imperceptible movement of the actor’s hand was interpreted as a request to move the out-of-reach cup closer to the actor so that she could complete the action (Figure 2). Notably, the type of grasp the participant observed and the one that was needed to complete the actor’s task were mismatched in all of the videos (i.e., a WHG performed by the actor vs. a PG required of the observer, and *vice versa*). As the participants were instructed to remain motionless throughout the task, the degree to which the motor system was activated provided an index of the CS activity elicited by action preparation. Moreover, as no explicit instructions were imparted to the participants, the experiment uncovered spontaneous tendencies to fulfill an implicit request embedded in a social interaction. This experiment was particularly enlightening in view of the fact that most studies typically ask participants to perform actions that are not associated with any meaningful behavior in real-world settings or utilize paradigms aiming to uncover dispositions formed during the execution of the experimental task itself (e.g., in imitation vs. complementary blocks) rather than spontaneous tendencies. Study results showed that a matching mechanism at the beginning of an action sequence turned into a complementary one as soon as the request for a reciprocal action became evident (*functional shift*). The muscle-specificity of MEP recordings highlighted the interplay between the initial tendency to resonate with what was observed and the subsequent inclination to implicitly prepare for a dissimilar complementary action (Figure 2).

At this point a new important question arose: at what point does this functional switch occur? A new experiment was designed in which TMS was delivered at *five* different timepoints corresponding to five kinematic landmarks characterizing the observed action (Sartori et al., 2013b,c). The most critical was the fourth (T_4_) timepoint when the actor’s hand trajectory began to significantly move toward the out-of-reach object. A TMS pulse was delivered precisely at that moment to investigate whether participants were able to predict the actor’s trajectory even before the action became explicit. The control condition that was designed consisted in the actor bringing her hand back to its initial position—with the out-of-reach object still visible in the foreground. The results showed that the participants quickly discriminated between an action driven by a social goal and one that was not, simply by observing the kinematic cues signaling the direction of the actor’s hand. These findings have direct implications with regard to action representation theories because they suggest that intention attribution (i.e., social vs. individual) is sensitive to kinematic constraints. As different types of intentional actions have distinctive motion signatures, observers appear to take note of precocious differences in kinematics during action observation in order to be able to predict the actor’s intentions (Kilner et al., 2007; Sartori et al., 2009, 2011a; Becchio et al., 2010, 2012a,b; Manera et al., 2011).

## A Working Memory Hypothesis

A dual process seems then to underlie joint actions: a low-level motor resonance analyzes and stores information on observed actions, while a high-level system would flexibly integrate our and others’ motor intentions and select the most appropriate response and time course to achieve joint goals (van Schie et al., 2008a). It can be hypothesized then that the hMNS’ function is similar to that of a working memory, although specifically tailored for action. Mirroring the responses of others might be useful to constantly track and monitor the interacting partners, and to support temporal coordination and action planning (Colling et al., 2013) while cognitive control systems come into play to distinguish self- and other-related representation, to inhibit unwanted imitative responses and to enforce self-generated actions (Brass et al., 2009; Cross et al., 2013). As in the case of the working memory, distinct elements would be kept on-line while others are being processed (Gibson, 2000). We therefore suggest an extension of the previous models of imitation control involving a cross-talk and a *simultaneous* activation of low- and high-level systems.

Complementary actions are the ideal way to test this topic. During complex social interaction/s, the individual needs to keep information relative to the observed action available while contemporaneously attempting to process a response. In this type of context, the mirror system may be involved in keeping action-related information on hold to enable other brain areas to extract the meaning of the action observed to achieve a joint goal. Notably, observing another person’s actions priming for an incongruent reaction can lead to a motor resonant response in the observer’s corresponding muscles as well as a simultaneous preparation in different effectors necessary for achieving a complementary response (Sartori et al., 2015). The relation between observed and executed actions seems then to be coordinated by a social associative memory which apparently matches some actions to their natural social responses regardless of who is actually performing the action (Chinellato et al., 2013). Under this model, there would be no difference between congruent, incongruent and complementary responses, as long as they have been associatively linked. In this vein, Catmur et al., (2007; 2008; 2009; see also Heyes, 2001, 2011; Cooper et al., 2013) have proposed that flexibility in action perception coupling may be gained thanks to associative sequence learning (i.e., the ASL theory) developed during social interactions. They strongly suggests that overlearned responses are able to modulate the motor priming effect: when a specific behavior is contingent on a non-matching behavior (e.g., extending the right hand when observing a right hand), an incongruent association is formed.

## Conclusion

The research outlined here shows that motor resonance elicited by action observation is modulated depending on its context: when an observed gesture is socially relevant (i.e., there is an implicit or explicit request) anticipatory complementary activations follow. The assumption that observing an action automatically triggers the inclination to imitate probably arose because most studies did not explicitly challenge the automaticity or flexibility of the visuomotor transformation process. The data outlined here have contributed to shedding light on the functioning of the human motor system in social contexts and on the types of social behavior frequently occurring in real-world settings. From a wider perspective, we can theorize that defining the conditions and the modalities by which motor resonant responses to action observation can be modulated may prove to have specific translational implications leading to the development of novel neuro-rehabilitation protocols for patients with localized lesions to cortical motor areas (e.g., ischemic stroke) and for pathologies such as autism (Hamilton, 2015). More distant horizons may include developing models of brain mechanisms underlying social interactions in the effort to endow artificial agents such as robots with the ability to perform meaningful complementary actions in response to observed actions.

### Conflict of Interest Statement

The authors declare that the research was conducted in the absence of any commercial or financial relationships that could be construed as a potential conflict of interest.
